# Longitudinal and Comparative Measures of Serum Chitotriosidase and YKL-40 in Patients With Idiopathic Pulmonary Fibrosis

**DOI:** 10.3389/fimmu.2022.760776

**Published:** 2022-02-10

**Authors:** Sebastian Majewski, Karolina Szewczyk, Hanna Jerczyńska, Joanna Miłkowska-Dymanowska, Adam J. Białas, Łukasz Gwadera, Wojciech J. Piotrowski

**Affiliations:** ^1^ Department of Pneumology, Medical University of Lodz, Lodz, Poland; ^2^ Department of Pathobiology of Respiratory Diseases, Medical University of Lodz, Lodz, Poland; ^3^ Central Scientific Laboratory (CoreLab), Medical University of Lodz, Lodz, Poland

**Keywords:** chitotriosidase, chitinase 1, CHIT1, chitinase 3-like-1, YKL-40, idiopathic pulmonary fibrosis, IPF, biomarker

## Abstract

**Background:**

Although chitin is absent in humans, chitinases are present in healthy subjects and show dysregulated expression in a variety of diseases resulting from abnormal tissue injury and repair responses. It was shown that chitotriosidase (chitinase 1/CHIT1) and structurally-related chitinase 3-like 1 protein (CHI3L1/YKL-40) play important roles in the pathobiology of idiopathic pulmonary fibrosis (IPF), however little is known about their longitudinal serum levels and relationship to clinical measures in IPF.

**Methods:**

The present study is the first to evaluate serial measurements of serum CHIT1 activity and YKL-40 concentrations in patients with IPF starting antifibrotic treatment and followed up for 24 months. In addition, baseline serum CHIT1 and YKL-40 were compared between patients with IPF and control subjects, and possible CHIT1 and YKL-40 relationships to longitudinal clinical assessments in IPF were explored.

**Results:**

Baseline serum CHIT1 activity and YKL-40 concentrations were significantly elevated in patients with IPF compared to control subjects and showed similar discriminatory ability in distinguishing IPF from controls. No significant differences between the median serum CHIT1 activity and YKL-40 concentration measured over a study follow-up were noted. We found significantly elevated baseline serum CHIT1 activity in the progressors compared with the stables in the first year, while significantly increased baseline serum CHIT1 activity was noted in the stables compared to the progressors in the second year. Additionally, we observed a significant negative correlation between a change in serum YKL-40 concentration and a change in forced vital capacity (FVC) % predicted (% pred.) in the stables subgroup, whereas, a change in serum CHIT1 activity correlated negatively with a change in FVC% pred. in the progressors subgroup.

**Conclusions:**

This explorative study findings add further evidence that CHIT1 and YKL-40 are upregulated in patients with IPF, and suggest that longitudinally stable serum CHIT1 activity and YKL-40 concentration levels may potentially be associated with the antifibrotic treatment response. In addition, our findings are supporting the possible role of CHIT1 and YKL-40 as candidate diagnostic and prognostic biomarkers in IPF. Further research is needed to validate present study findings.

## Introduction

Chitin is a polysaccharide polymer abundantly present in the environment. It is a structural constituent of the bacterial and fungal cell walls, the exoskeletons of crustaceans, the sheaths of parasitic nematodes, and the lining of the digestive tract of many insects. Those species possess several hydrolytic enzymes - chitinases, responsible for chitin metabolism, and are involved in many physiologic processes including growth and development (1). True chitinases, including chitotriosidase (chitinase 1/CHIT1) are active enzymes able to bind and degrade chitin (2). Many organisms, in addition to true chitinases, produce various structurally related chitinase-like proteins (CLPs), which express several regulatory functions but are lacking chitinolytic activity (1, 3). One of the most widely described CLPs is a chitinase 3-like 1 (CHI3L1/YKL-40). In humans, despite the absence of endogenous chitin, both true chitinases and CLPs have been identified. Nevertheless, their biological roles are poorly understood and have only recently begun to be revealed. A growing body of evidence suggests various regulatory functions of chitinases and CLPs in immune response regulation, inflammation, tissue damage, and tissue remodeling in both health and disease (1, 4–7).

Idiopathic pulmonary fibrosis (IPF) results from repeated micro-injuries to alveolar epithelium, caused by the exposure to various noxious stimuli, leading in genetically predisposed individuals to subsequent dysfunction of the alveolar epithelial cells (AECs), aberrant healing, and diffuse parenchymal fibrosis in the final stage of the pathogenic process (8–12). As consequence patients with IPF develop progressive dyspnea, the gradual decline of lung function, physical activity limitation, impairment of quality of life, and premature death with a median survival of 3 to 5 years (8, 13). Recently, antifibrotic therapy has been shown to modify the natural history of IPF, improve disease outcomes and survival (14–19).

It has been demonstrated previously that chitin as an abundantly present environmental polymer can be inhaled into the lungs and accumulate in patients with various respiratory conditions, including IPF, which in turn raises the possibility that chitin altered clearance may contribute to inflammatory and fibrotic pathways in the setting of lung diseases characterized by lung epithelium dysfunction (20–22). Moreover, studies demonstrated increased CHIT1 activity levels in serum and/or lung samples of patients with IPF (23, 24) and more recently upregulation of CHIT1 in single-cell transcriptomes of novel subpopulation pulmonary fibrosis-specific macrophages (25, 26). In addition, previous research revealed overexpression of YKL-40 in serum and/or lung samples in patients with IPF (27–30) and the regulatory role of YKL-40 in the development and progression of pulmonary fibrosis (31). Nevertheless, there is a knowledge gap whether an association between the amount of accumulated chitin in the lung and levels of chitinases and CLPs exists, and if any of these two or both can explain disease initiation, severity or progression. Therefore, the complex mechanisms and roles of chitinases and CLPs interactions and their possible impact on pathobiology of IPF remain to be elucidated.

To date, relatively little is known about circulating CHIT1 and YKL-40 and their associations with clinical assessments in patients with IPF. Furthermore, no previous research has investigated the longitudinal changes in serum CHIT1 activity and YKL-40 concentration levels in patients with IPF receiving antifibrotics.

In the present study, we aimed to evaluate the longitudinal changes in circulating CHIT1 and YKL-40 in patients with IPF. In addition, we compared baseline serum CHIT1 activity and YKL-40 concentrations in IPF and control subjects and explored the possible relationship of serum CHIT1 and YKL-40 to serial clinical measures in a cohort of patients with IPF starting antifibrotic therapy.

## Materials and Methods

### Study Population

We retrospectively enrolled 25 patients with IPF (14 males (56%), with a mean age of 68.5 ± 8.03 years) qualified for antifibrotic therapy and regularly monitored at the Department of Pneumology, Medical University of Lodz. The eligibility criteria of participant enrollment included a confident IPF diagnosis confirmed by a multidisciplinary team according to the international guidelines (13) and a follow-up period of at least 2 years during antifibrotic therapy. The exclusion criteria included a switch of an antifibrotic drug due to any reason over a study follow-up or a follow-up period shorter than 2 years. A group of 20 age-matched volunteers with no previous history of respiratory diseases was recruited as a control group. The IPF cohort included 18 patients qualified for the treatment with pirfenidone and 7 patients qualified for the treatment with nintedanib. The study protocol was reviewed and approved by the Ethics Committee of the Medical University of Lodz (approval number RNN/66/17/KE, date 14.03.2017). The study was conducted according to the Declaration of Helsinki principles and all study participants gave written informed consent before the start of any study-related procedures.

### Methods

Enrolled patients with IPF underwent serial peripheral blood sampling and clinical assessments consisting of pulmonary function tests (PFTs), including spirometry and single-breath transfer factor of the lung for carbon monoxide (T_L,CO_) measurements, and a functional assessment using a six-minute walk test (6MWT). All study procedures were performed at baseline and after 6, 12, 18, and 24 months of antifibrotic treatment. Spirometry and T_L,CO_ measurements were performed using the Lungtest 1000 system (MES, Cracow, Poland) according to ATS/ERS standards (32, 33). Forced expiratory volume in 1 second (FEV_1_), forced vital capacity (FVC), and measurements of T_L,CO_ corrected for hemoglobin concentration were recorded. For the expression of PFTs results as percent of the predicted values (% pred.), the Global Lung Function Initiative (GLI) reference values were adopted. The composite physiologic index (CPI) score was calculated for each of the IPF patients according to the following formula, as described previously (34): 91.0−(0.65 × T_L,CO_% pred.)−(0.53 × FVC% pred.) + (0.34 × FEV_1_% pred.). The control subjects underwent a peripheral venous blood sampling and spirometry at baseline only.

### Blood Samples Processing

Peripheral venous blood samples were drawn into serum separator tubes (SST) (BD Dickinson) and left at room temperature for clotting for 30 minutes. Then, the samples were centrifuged for 15 minutes at 1000×g and stored at −80°C for further assessment.

### Chitotriosidase Assay

Commercially available CycLex Chitotriosidase Fluorometric Assay Kit (Medical&Biological Laboratories CO., LTD., Nagano, Japan) was used to measure chitotriosidase activity in serum samples in a similar way as previously reported by our group (35). Briefly, chitotriosidase activity was measured using 4-methylumbelliferyl-β-N-N’-N”-triacetylchitotrioside (4-MUC) as a fluorogenic glycanase substrate with a final concentration of 0.02 mM. The enzyme reaction was initiated by the addition of 95 µl of Fluoro-Substrate solution (4-MUC and Assay Buffer diluted in water) to 5 µl of sample. 4-MUC was hydrolyzed by chitotriosidase present in the sample, producing 4-methylumbelliferone (4-MU) molecule. Fluorescence of 4-MU was measured for 50 minutes at 4 minutes intervals using VICTOR X4 Multilabel plate reader (Perkin Elmer Inc, Waltham, MA, USA) with excitation at 355 nm and emission at 460 nm. Chitotriosidase activity was determined using the slope of the 4-MU standard curve as the conversion factor and was expressed as nanomoles of substrate hydrolyzed per milliliter per hour (nmol/ml/h). Reported values are the average of three measurements.

### YKL-40 Assay

Serum YKL-40 concentrations were measured using commercially available enzyme-linked immunosorbent assay (Biorbyt Ltd., Cambridge, UK) according to the manufacturer’s instructions. An assay sensitivity was 10 pg/ml and detection range 62.5 pg/ml – 4000 pg/ml. Each sample was tested in duplicate. Reported values are the average of two measurements.

### Definition of IPF Progression

The composite definition of IPF progression was described as ≥10% absolute decline in FVC% pred. and/or ≥15% absolute decline in T_L,CO_% pred. and/or ≥50 meters decline in 6MWT distance within 12 months period of antifibrotic therapy (12 months vs baseline and 24 months vs 12 months study timepoint). Patients fulfilling the above criteria were classified as progressors, whereas the others were classified as stables.

### Statistical Analysis

The data were analyzed using a GraphPad Prism 9 (GraphPad Software, La Jolla, San Diego, CA, USA) except for receiver-operating characteristic (ROC) curve analysis where NCSS 2021 Statistics Software version 21.0.2 (NCSS, LLC. Kaysville, Utah, USA) was used. The Shapiro-Wilk test was used for the assessment of normality of data distribution. The continuous data are expressed as mean with standard deviation (SD) for normally distributed data or as median with interquartile range (IQR) for nonparametric data. Categorical variables are presented as either a percentage of the total or numerically, as appropriate. Data were analyzed using paired t-test, Wilcoxon signed-rank test, unpaired t-test, or the Mann-Whitney U test, depending on data normality and homogeneity of variance. For comparison of multiple groups of the paired sample, we used the Friedman test with Dunn’s correction for nonparametric data, whereas for parametric data we applied one-way repeated measures ANOVA with the Geisser-Greenhouse correction. The Spearman correlation coefficient was used to evaluate correlations. The ROC curves were constructed to evaluate the discriminating capability of CHIT1 and YKL-40 to differentiate patients with IPF from control subjects. Cut-off levels for serum CHIT1 activity and YKL-40 concentration were determined using the Youden index. We performed an analysis of possible associations between longitudinal changes in serum CHIT1 activity and YKL-40 concentration and longitudinal changes in FVC% pred., T_L,CO_% pred., and 6MWT distance. All changes were evaluated separately in the subgroup of patients with stable and progressive disease over the first and the second year of study follow-up and were counted as the difference in values at 12 months vs baseline and 24 months vs 12 months study timepoint, respectively. Changes in serum CHIT1 activity, YKL-40 concentration and 6MWT distance were expressed in % of relative change, whereas, the changes in FVC% pred. and T_L,CO_% pred. were expressed as the absolute change of % predicted values. The significance was accepted at p<0.05.

## Results

### Baseline Characteristics of Study Participants

The characteristics of the study population are presented in **Table 1**. The mean age of patients with IPF was 68.5 ± 8.03 years, and the majority of them (72%) were active or past smokers with a median of 20 pack-years smoking exposure. The median disease duration before the start of antifibrotic treatment was more than 1.5 years. The PFTs data at baseline in our IPF patients showed moderate lung function impairment with mean FVC% pred. of 73.3 ± 19.1% and mean T_L,CO_% pred. of 52.5 ± 13.2%.

**Table 1:** Baseline characteristics of the study population.

	Controls	IPF
Number of subjects	20	25
Sex (male/female)	10/10	14/11
Age (years), mean (SD)	68.40 (6.11)	68.53 (8.03)
Smoking history (pack-years), median (IQR)	2.55 (0-36.13)	20.00 (0-30)
Smoking status		
- never smokers, n (%)	9 (45.00%)	7 (28.00%)
- ex-smokers, n (%)	3 (15.00%)	17 (68.00%)
- current smokers, n (%)	8 (40.00%)	1 (4.00%)
CPI score, mean (SD)	N/A	69.64 (7.17)
Time since diagnosis (years), median (IQR)	N/A	1.58 (0.59-3.65)
FVC (l), mean (SD)	3.88 (1.21)	2.58 (0.85)***
FVC (% of predicted), mean (SD)	111.30 (20.73)	73.35 (19.11)****
T_L,CO_ (mmol/min/kPa), mean (SD)	N/A	3.91 (1.12)
T_L,CO_ (% of predicted), mean (SD)	N/A	52.50 (13.22)
6MWT (meters), mean (SD)	N/A	388.00 (102.10)

***p < 0.001; ****p < 0.0001.

IPF, idiopathic pulmonary fibrosis; CPI, composite physiologic index; FVC, forced vital capacity; T_L,CO_, transfer factor of the lung for carbon monoxide; 6MWT, six-minute walk test.

### Baseline Serum CHIT1 Activity and YKL-40 Concentration

**Figure 1:**
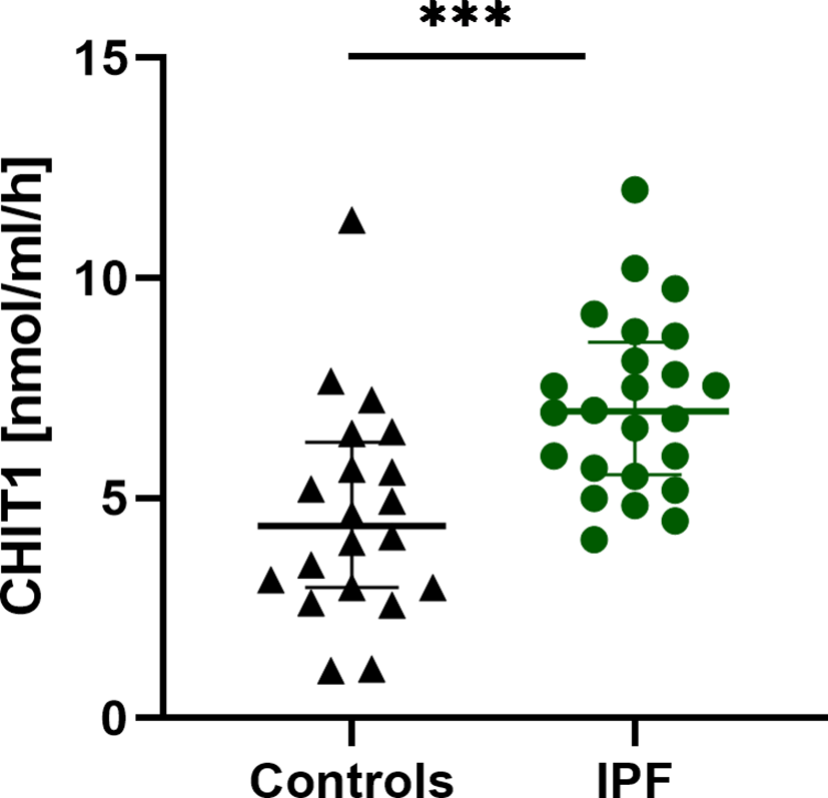
Baseline serum CHIT1 activity in patients with IPF (n=25) and control subjects (n=20). Median and interquartile range (IQR) are depicted. ***p < 0.001. CHIT1, chitotriosidase; IPF, idiopathic pulmonary fibrosis.

**Figure 2:**
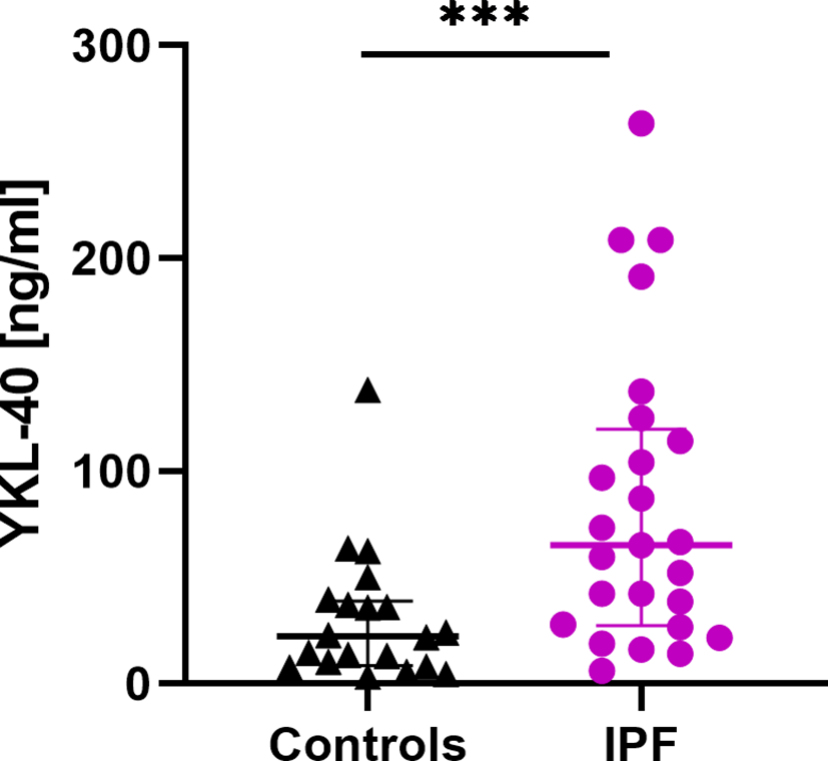
Baseline serum YKL-40 concentrations in patients with IPF (n=25) and control subjects (n=20). Median and interquartile range (IQR) are depicted. ***p < 0.001. YKL-40, chitinase 3-like-1; IPF, idiopathic pulmonary fibrosis.

Serum CHIT1 activity and YKL-40 concentrations were measurable in all subjects studied. We noted significantly elevated baseline serum CHIT1 activity in patients with IPF (6.97 (5.54-8.54) nmol/ml/h) compared to controls (4.37 (2.97-6.27) nmol/ml/h; p<0.001), see **Figure 1**. Baseline serum YKL-40 concentration was also significantly increased in patients with IPF compared to control subjects (65.20 (27.25-119.60) ng/ml vs. 22.35 (8.73-38.93) ng/ml; p < 0.001), see **Figure 2**.

**Figure 3:**
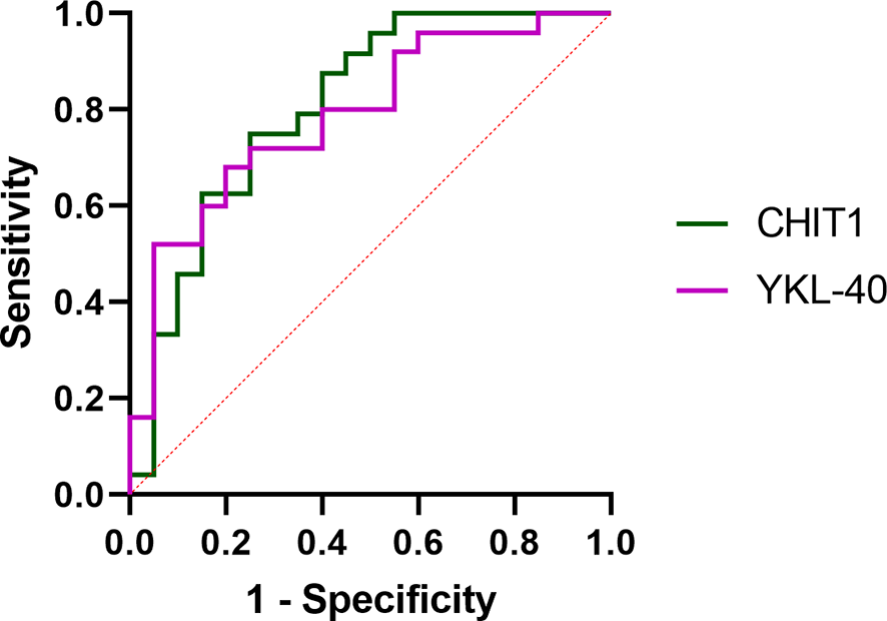
ROC curve analysis of serum CHIT1 activity and YKL-40 concentration to distinguish IPF from control subjects. ROC, receiver operating characteristic; CHIT1, chitotriosidase; YKL-40, chitinase 3-like 1.

ROC curves were constructed to evaluate the discriminating capability of serum CHIT1 and YKL-40 to differentiate IPF subjects from controls, see **Figure 3**. Baseline serum CHIT1 activity and YKL-40 concentration had a similar discriminatory ability to differentiate patients with IPF from controls (AUC value of 0.806; p=0.00001 for CHIT1 and AUC value 0.788; p=0.0001 for YKL-40). More detailed information including the optimal cut-off values, sensitivity, and specificity are presented in **Table 2**.

**Table 2:** Discriminating capability and cut-off values of baseline serum CHIT1 and YKL-40 by ROC curve analysis distinguishing IPF from controls.

	CHIT1	YKL-40
AUC	0.806	0.788
95% CI	0.6231-0.9056	0.6118-0.8897
p-value	0.00001	0.00001
Cut-off value	5.67 nmol/ml/h	40.90 ng/ml
Sensitivity	75.00%	68.00%
Specificity	75.00%	80.00%
PPV	78.26%	80.95%
NPV	71.43%	66.67%

CHIT1, chitotriosidase; YKL-40, chitinase 3-like-1; AUC, area under the curve; CI, confidence interval; PPV, positive predictive value; NPV, negative predictive value.

### Longitudinal Associations of Serum CHIT Activity and YKL-40 Concentration With Clinical Measures in Patients With IPF

The longitudinal changes in PFTs, 6MWT distance, serum CHIT1 activity and YKL-40 concentrations in the IPF cohort evaluated in 6-months intervals are presented in **Table 3**. Over a study follow-up, the mean FVC and T_L,CO_ were relatively preserved. The mean 6MWT distance decreased significantly after 24 months of study duration. No significant differences between the median serum CHIT1 activity and YKL-40 concentration levels measured in the consecutive study timepoints were noted, see **Table 3**.

**Table 3:** Longitudinal changes in PFTs, 6MWT, and serum CHIT1 and YKL-40 in patients with IPF.

	Baseline	6 months	12 months	18 months	24 months
FVC (l), mean (SD)	2.58 (0.85)	2.61 (0.79)	2.52 (0.83)	2.53 (0.82)	2.51 (0.80)
FVC% pred., mean (SD)	73.35 (19.11)	73.82 (18.00)	72.55 (19.03)	72.97 (20.35)	72.84 (19.23)
T_L,CO_ (mmol/min/kPa), mean (SD)	3.91 (1.12)	3.73 (1.19)	3.25 (1.25)^‡‡‡‡§§^	3.16 (1.29)^^^^***	3.28 (1.20)^###^
T_L,CO_% pred., mean (SD)	52.50 (13.22)	50.73 (15.24)	43.62 (15.82)^‡‡‡§§^	45.91 (21.12)	49.13 (20.94)
6MWT (meters), mean (SD)	388.00 (102.10)	405.10 (102.2)	367.60 (128.3)	372.40 (103.6)	339.40 (114.4)*
CHIT1 (nmol/ml/h), median (IQR)	6.97 (5.54-8.54)	7.38 (4.78-9.07)	6.90 (5.99-8.83)	6.78 (5.47-7.50)	7.09 (5.48-8.43)
YKL-40 (ng/ml), median (IQR)	65.20 (27.25-119.60)	55.70 (27.35-114.20)	53.20 (27.95-122.50)	79.80 (32.75-119.80)	54.70 (32.75-169.50)

Baseline vs 12 months, ^‡‡‡^p < 0.001, ^‡‡‡‡^p < 0.0001; Baseline vs 18 months, ^^^^p < 0.0001; Baseline vs 24 months, ^###^p < 0.001; 6 vs 12 months, ^§§^p < 0.01; 6 vs 18 months, ***p < 0.001; 6 vs 24 months, *p < 0.05.

IPF, idiopathic pulmonary fibrosis; PFTs, pulmonary function tests; FVC, forced vital capacity; T_L,CO_, transfer factor of the lung for carbon monoxide; 6MWT, six-minute walk test; CHIT1, chitotriosidase; YKL-40, chitinase 3-like-1.

Analysis of possible relationships between serum CHIT1 activity and YKL-40 concentration and clinical measures in patients with IPF revealed a positive correlation between serum YKL-40 and age of IPF subjects at baseline (r=0.56, p<0.01, **Table S1**), 6 months (r=0.48, p<0.05, **Table S2**), 12 months (r=0.45, p<0.05, **Table S3**), and 18 months of antifibrotic treatment (r=0.41, p<0.05, **Table S4**). Similarly, serum YKL-40 concentrations correlated positively with CPI score at baseline (r=0.54, p<0.01, **Table S1**), and 6 months of treatment (r=0.55, p<0.01, **Table S2**). Moreover, we noted a positive correlation between serum YKL-40 concentrations and FVC% pred. at 24 months of treatment (r=0.40, p<0.05, **Table S5**). In addition, we observed a negative correlation between serum YKL-40 concentrations and 6MWT at 6 months of treatment (r=-0.45, p<0.05, **Table S2**). Baseline serum CHIT1 activity correlated negatively with FVC absolute values (r=-0.49, p<0.05, **Table S1**). All possible correlations between serum CHIT1 and YKL-40 and clinical measures in the IPF cohort are presented in **Supplementary Tables S1**–**S5** (**Supplementary Material**).

### Longitudinal Changes in Serum CHIT1 Activity and YKL-40 Concentration According to the Disease Progression Assessment of Patients With IPF

**Figure 4:**
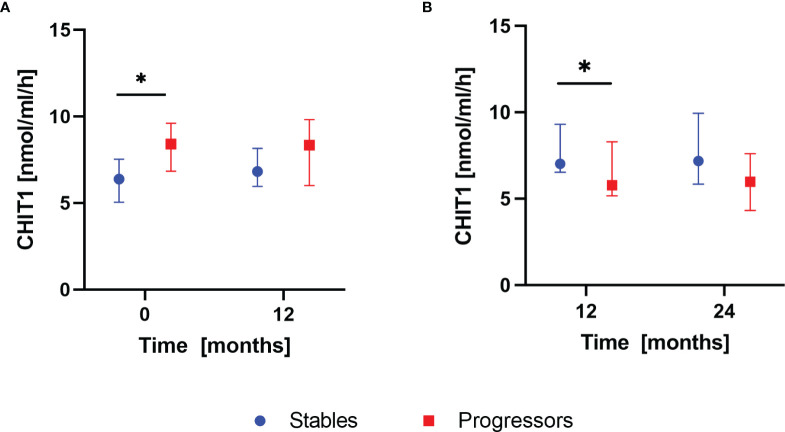
Serum CHIT1 activity in IPF patients with stable and progressive disease over the first and the second year of antifibrotic therapy. Data are presented as the median and interquartile range (IQR). Panels showing: **(A)** serum CHIT1 activity measured in stables (n=16) and progressors (n=9) at baseline and 12 months, **(B)** serum CHIT1 activity measured in stables (n=15) and progressors (n=10) at 12 and 24 months; *p < 0.05; CHIT1, chitotriosidase.

**Figure 5:**
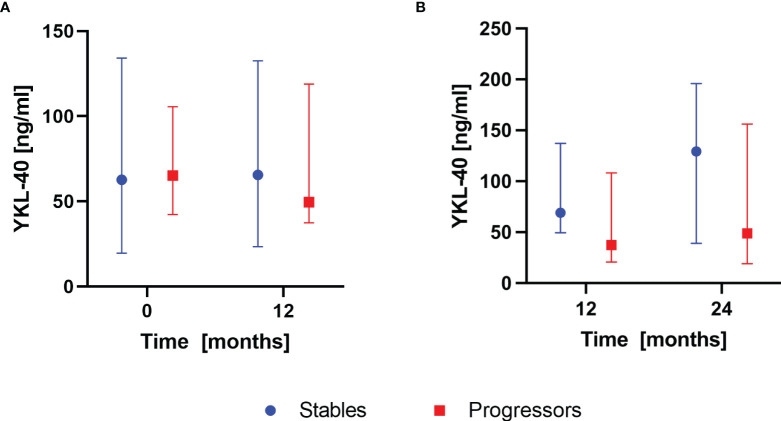
Serum YKL-40 concentrations in IPF patients with stable and progressive disease over the first and the second year of antifibrotic therapy. Data are presented as the median and interquartile range (IQR). Panels showing: **(A)** serum YKL-40 concentrations measured in stables (n=16) and progressors (n=9) at baseline and 12 months, **(B)** serum YKL-40 concentrations measured in stables (n=15) and progressors (n=10) at 12 and 24 months. YKL-40, chitinase 3-like-1.

According to the composite definition of IPF progression, after the first year of antifibrotic therapy, 16 subjects were classified as stables and 9 subjects were classified as progressors. During the second year, 15 subjects were classified as stables and 10 subjects were classified as progressors. None of the patients experienced progression consecutively over both 12-months follow-up periods. Analysis of baseline serum CHIT1 activity and YKL-40 concentration in subgroups of patients with stable and progressive disease within the first year of antifibrotic therapy revealed significantly elevated serum CHIT1 activity in the progressors compared with the stables (8.40 (6.83-9.61) nmol/ml/h vs 6.39 (5.04-7.54) nmol/ml/h; p<0.05), see **Figure 4A**. Over the second year of antifibrotic therapy, we observed significantly increased serum CHIT1 activity in patients with stable disease (7.02 (6.53-9.30) nmol/ml/h) in comparison with the progressors subgroup (5.78 (5.17-8.30) nmol/ml/h), see **Figure 4B**. No significant differences were observed in serum YKL-40 concentrations between patients with stable or progressive disease over a study follow-up, see **Figure 5**. Additionally, no significant dynamic changes of neither serum CHIT1 nor YKL-40 in the stables and progressors subgroups were noted, see **Figures 4**, **5**. Single patient dynamic changes of serum CHIT1 activity and YKL-40 concentration levels in the stables and progressors over a study follow-up are shown in **Supplementary Figures S1**, **S2** (**Supplementary Material**).

### Associations of Longitudinal Changes in Serum CHIT1 Activity and YKL-40 Concentration and Changes in PFTs and 6MWT in Subgroups of Patients With IPF

**Figure 6:**
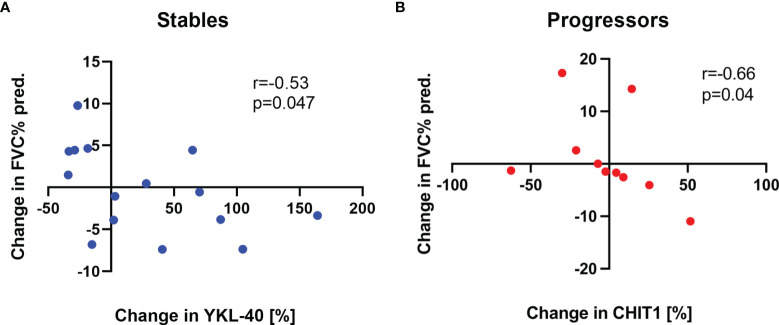
Associations of changes in serum CHIT1 activity and YKL-40 concentration with changes in FVC% pred. in patients with the stable and progressive disease over the second year of antifibrotic treatment. Panels showing: **(A)** a correlation between changes in serum YKL-40 concentration levels and changes in FVC% pred. in the stables subgroup (n=15), **(B)** a correlation between changes in serum CHIT1 activity levels and changes in FVC% pred. in the progressors subgroup (n=10), FVC, forced vital capacity; CHIT1, chitotriosidase; YKL-40, chitinase 3-like-1.

In the first year of antifibrotic therapy, no significant correlations between longitudinal changes in serum CHIT1 activity and YKL-40 concentration and longitudinal changes in FVC% pred., T_L,CO_% pred., and 6MWT distance were observed in any of the subgroups of IPF patients. In the second year of antifibrotic therapy, we observed a significant negative correlation between a change in serum YKL-40 concentration levels and a change in FVC% pred. (r=-0.53, p<0.05), see **Figure 6A** in the stables subgroup. Moreover, in the progressors subgroup, a change in serum CHIT1 activity levels correlated negatively with a change in FVC% pred. (r=-0.66, p<0.05), see **Figure 6B**. All possible correlations of longitudinal changes in serum CHIT1 activity and YKL-40 concentration levels with changes in FVC% pred., T_L,CO_% pred., and 6MWT distance in patients with stable and progressive disease are presented in **Supplementary Tables S6**–**S9** (**Supplementary Material**).

## Discussion

This study investigated serial changes in serum CHIT1 and YKL-40 for up to 24 months in a cohort of patients with IPF receiving antifibrotic treatment. In addition, baseline serum CHIT1 activity and YKL-40 concentration were compared between patients with IPF and control subjects, and possible CHIT1 and YKL-40 relationships to longitudinal clinical assessments in IPF were explored. The main findings of the present study are that baseline serum CHIT1 activity and YKL-40 concentrations are significantly increased in IPF compared with control subjects and both show similar discriminatory ability in distinguishing IPF patients from controls. No significant differences between serial measurements of serum CHIT1 activity and YKL-40 concentration levels over 24 months of study follow-up were noted. Longitudinal data revealed that baseline serum CHIT1 activity was most clearly distinguishing patients with progressive and stable disease, however, results were contradictory in the first and the second year of study follow-up. No significant dynamic changes of serum CHIT1 or YKL-40 in patients with progressive or stable disease were noted. In addition, a significant inverse correlation between a change in serum YKL-40 concentration and a change in FVC% pred. was observed in the stables subgroup of patients, while a significant inverse correlation between a change in serum CHIT1 activity and a change in FVC% pred. in the progressors subgroup was noted. Overall, our study findings add further evidence that CHIT1 and YKL-40 are upregulated in patients with IPF, and suggest that their longitudinally stable serum levels may potentially be associated with the antifibrotic treatment response. Additionally, the study results are supporting the possible role of CHIT1 and YKL-40 as candidate diagnostic and prognostic biomarkers in IPF. Further research is necessary to validate our exploratory findings and to understand the precise roles of chitinases in biological functions in IPF.

Chitinases and CLPs can be found in the circulation and tissues of both healthy subjects and patients with various acute and chronic disorders characterized by inflammation and remodeling (1, 4–7). However, their precise roles in health and disease are poorly understood because no endogenous substrate for chitinases or CLPs has been identified in humans. CHIT1 is the best characterized true chitinase from a biological and clinical perspective (36) and is the most prominent chitinase in human lung and circulation (37). CHIT1 is secreted by activated macrophages, yet other sources of CHIT1, including neutrophils and structural cells, were identified (36, 38). YKL-40 belongs to the mammalian CLPs family members, which bind chitin with high affinity, but lack chitinolytic activity. YKL-40 is produced by various cell types, including macrophages, neutrophils, monocytes, and several structural cells (39, 40).

Macrophages have been recognized to play a significant role in the pathobiology of IPF. Depending on the local microenvironments, macrophages can be polarized to classically activated (M1) or alternatively activated (M2) phenotypes. In general, M1 macrophages are responsible for wound healing after AEC injury, while M2 macrophages are designated to resolve wound healing processes or terminate inflammatory responses in the lung (41). Moreover, macrophages are known to play pivotal roles in immune regulation. It is of note, that previous studies suggested an active role of both CHIT1 and YKL-40 in monocyte to macrophage transition and polarization which is supporting their contribution in innate and acquired immune responses and involvement in maintaining the homeostasis in the immune system (42). Recent studies have demonstrated increased CHIT1 expression in the single-cell transcriptomes of macrophage subpopulations in IPF (25, 26). It is well known, that transforming growth factor-β1 (TGF-β1) is a key regulator of pulmonary fibrosis as well as other fibrotic diseases of various organs. YKL-40 is known to drive inflammatory pathways while preventing apoptosis and inducing fibrosis through molecules like TGF-β1. It also plays an essential role in the induction of alternative macrophage activation (43). Taken together, it can be speculated that activated macrophages in patients with IPF are responsible for the upregulation of CHIT1 and YKL-40 which in turn may contribute to the progression of lung fibrosis. Nevertheless, the exact biological roles, possible interactions, and contributions of CHIT1 and YKL-40 in the pathogenesis of IPF are not clearly defined and remain to be elucidated.

To the best of our knowledge, the present study is the first to investigate the longitudinal changes in circulating CHIT1 and YKL-40 in patients with IPF receiving antifibrotics. Herein, we demonstrated significantly increased baseline serum CHIT1 activity and YKL-40 concentration levels in IPF compared with control subjects. These findings are in line with some data from the previous research on the topic, however, published reports regarding CHIT1 activity and YKL-40 concentrations in IPF are only a few and some results are contradictory (23, 24). One previous study evaluating CHIT1 activity levels in patients with interstitial lung disease (ILD) showed that serum CHIT1 activity was only elevated in sarcoidosis patients, while in patients with IPF and patients with systemic sclerosis-associated ILD (SSc-ILD) serum CHIT1 activity levels were not different compared to controls. However, the same study results revealed significantly increased CHIT1 activity levels in bronchoalveolar lavage fluid (BALf) of sarcoidosis and IPF patients than in controls, suggesting compartment-specific regulation of CHIT1 (23). On the contrary, more recent research reported elevated CHIT1 activity in both serum and induced sputum obtained from IPF patients compared to controls and demonstrated overexpression of CHIT1 in BALf macrophages of IPF patients (24). Our research results support the shreds of evidence that CHIT1 is upregulated in IPF. Moreover, our study ROC curve analysis showed a discriminatory capability for serum CHIT1 activity in distinguishing IPF from controls using a cut-off value of 5.67 nmol/ml/h that supports serum CHIT1 potential as a diagnostic biomarker in IPF.

Our finding of increased serum YKL-40 concentrations in IPF compared with controls is in complete agreement with the previous clinical research studies in the ILD field reporting elevated YKL-40 concentrations in serum and/or BALf of patients with IPF (27–30), sarcoidosis (44, 45), or connective tissue disease-associated ILD (CTD-ILD) (29, 46–49). It has been also shown that high serum and BALf YKL-40 concentrations are associated with poor survival in patients with IPF and hypersensitivity pneumonitis (HP) (28, 30). We found, in agreement with others, a correlation between serum YKL-40 and the age of IPF patients (30, 50, 51). In contrast with the previous research, we have not confirmed a sporadically reported correlation between baseline serum YKL-40 and T_L,CO_ which was demonstrated in IPF (27), sarcoidosis (44, 45), and CTD-ILD (46). Taken together, our study data support the evidence that YKL-40 is upregulated in IPF. Moreover, the ROC curve analysis showed a discriminatory capability for serum YKL-40 in distinguishing IPF from controls using a cut-off value of 40.9 ng/ml that supports serum YKL-40 as a potential biomarker of an early diagnosis in IPF.

We observed relatively stable serially measured CHIT1 activity and YKL-40 concentrations over a study follow-up. To date, results of serial measurements of serum CHIT1 activity in IPF have not been reported. Although, it has been demonstrated that serial measurements of serum CHIT1 correlate with clinical symptoms, chest radiographs, and lung function in sarcoidosis (52) and may have the potential as high specificity biomarker of extrapulmonary manifestations of the disease (53). Only one previous study reporting serial measurements of YKL-40 in IPF patients not receiving antifibrotics showed that serum YKL-40 remains remarkably stable over time despite disease progression (29). We speculate that our novel finding of longitudinally stable serum CHIT1 and YKL-40 in patients with IPF over 24 months may potentially be associated with the antifibrotic treatment response. However, this novel exploratory finding needs confirmation in further studies performed in larger cohorts of patients.

The composite definition of IPF progression in our study included assessment of changes in physiologic (FVC and T_L,CO_), and functional (6MWT) markers of disease severity. An absolute decline in FVC of ≥10% or T_L,CO_ of ≥15% over 6 to 12 months has been regarded as clinically important and is frequently used to describe a significant disease progression (54). Moreover, the substantial evidence demonstrates that ≥10% decline in FVC is associated with a significant increase in mortality in IPF (55–58). Data regarding changes in T_L,CO_ as a clinical predictor of outcomes in IPF are inconclusive, nevertheless, studies show that trends in T_L,CO_ levels might provide important information for determining mortality (57). The longitudinal variation in the 6MWT distance has been used to reflect the disease status and progression (59–61), prognosis prediction (62) and has been shown to outweigh other predictors of mortality in IPF (63). A threshold for minimum clinically important difference value for 6MWT has been suggested as 24-45 meters or more (64). It is likely that the composite definition of disease progression used in our study, including both physiologic, and functional measures, reflects more broadly a clinically significant deterioration of patients with IPF in routine clinical practice. Our study longitudinal data revealed that baseline serum CHIT1 activity was most clearly distinguishing patients with the progressive and stable disease according to the composite definition of IPF progression. Nevertheless, the obtained results were contradictory between the first and the second year of patients’ follow-up. Mechanistic studies demonstrated that CHIT1 enhances TGF-β1-stimulated fibrotic cellular and tissue responses and TGF-β1 signaling, which suggests that CHIT1 is a fibrogenic modifier contributing to the pathogenesis of pulmonary fibrosis (65, 66). Therefore, increased CHIT1 expression may be associated with the disease progression in IPF. Interestingly, it was shown that inhibition of CHIT1 has more favorable therapeutic effects than nintedanib and comparable therapeutic efficiency to pirfenidone in the bleomycin-induced pulmonary fibrosis model (24, 67). CHIT1 is recently considered as a novel therapeutic target in IPF and the first-in-class CHIT1 inhibitor is currently studied as a potential treatment for IPF (68, 69). Based on our study’s novel finding that baseline serum CHIT1 activity could discriminate stables from progressors, we believe that CHIT1 could be useful as a potential biomarker of prognosis for the clinical practice in IPF, however further studies are warranted to confirm the present study results.

Although baseline serum CHIT1 activity and YKL-40 concentration levels were not associated with pulmonary function in our study cohort, we noted a significant relationship between a change in serum levels of CHIT1 activity and YKL-40 concentration and a change in FVC% pred. over study follow-up. Interestingly, a change in serum YKL-40 concentration level was inversely correlated with a change in FVC% pred. in the stables subgroup, while a change in serum CHIT1 activity level was inversely correlated with a change in FVC% pred. in the progressors subgroup of patients with IPF. No previous clinical studies in IPF reported similar findings, and no clear explanation for such observation is arising from the literature. We believe, these aforementioned exploratory findings underscore the complex biological roles of CHIT1 and YKL-40 as modulators of pathogenic mechanisms in IPF. It has been demonstrated previously that YKL-40 has complex roles in the development and progression of pulmonary fibrosis and its regulation must be carefully balanced. In an animal model of bleomycin-induced pulmonary fibrosis, YKL-40 played a protective role in injury by ameliorating inflammation and cell death, and a profibrotic role in the repair phase by augmenting alternative macrophage activation, fibroblast proliferation, and matrix deposition. In other words, downregulation of YKL-40 during the initial injury phase can result in subsequent, exaggerated fibrosis, while upregulation of YKL-40 in the fibrosis phase drives excessive deposition of collagen and other extracellular matrix proteins (31).

Despite the novel findings of the present study, it has several limitations. First, the sample size is relatively small which might lead to either under or overestimation of the observed effects. However, as this was an exploratory and not confirmatory study, the sample size estimation and power analysis were not calculated. Secondly, a selection bias due to the lack of patients with more advanced IPF in our cohort could have influenced the obtained results. It is of note, that patients with advanced IPF (FVC<50% of pred. and T_L,CO_<30% of pred.) are not eligible to receive reimbursed antifibrotics in our country, therefore, they could not be enrolled in the study. Thirdly, to answer the question of whether the stable serum levels of chitinases during antifibrotic therapy noted in our study are associated with the treatment response we would need a longitudinal comparison with the cohort of patients with IPF not receiving antifibrotics. Due to ethical concerns, such study construction was not feasible. Lastly, the present study has not included any survival analysis, which potentially limits the evaluation of the prognostic ability of CHIT1 and YKL-40 in IPF patients treated with antifibrotics. However, to study longitudinal changes in serum CHIT1 and YKL-40 only subjects with complete study timepoints had to be included in the analysis. Regardless of the above shortcomings, our study data substantiate the current knowledge on the longitudinal expression of CHIT-1 and YKL-40 in IPF. Additional studies planned in larger patient cohorts are warranted to validate our study results and to evaluate the biological significance of our findings.

In short, there are many gaps in our current knowledge of the biological roles of chitinases and CLPs in the pathobiology of IPF. Our study supports the evidence that CHIT1 and YKL-40 are upregulated in patients with IPF compared with controls and their serum measurements may offer a tool for early diagnosis of IPF. In addition, the present study’s longitudinal data showed for the first time that serum CHIT1 activity and YKL-40 concentration are stable in IPF patients treated with antifibrotics and may have the potential as candidate prognostic biomarkers in IPF clinical practice.

## Conclusions

In conclusion, the present study findings add further evidence that CHIT1 and YKL-40 are upregulated in patients with IPF, and suggest that longitudinally stable serum CHIT1 activity and YKL-40 concentrations may potentially be associated with the antifibrotic treatment response. In addition, our findings are supporting the possible role of CHIT1 and YKL-40 as candidate diagnostic and prognostic biomarkers in IPF. Further longitudinal and mechanistic studies are required to fully understand the precise roles of chitinases and CLPs in the biological processes of IPF.

## Data Availability Statement

The original contributions presented in the study are included in the article/**Supplementary Material**. Further inquiries can be directed to the corresponding author.

## Ethics Statement

The studies involving human participants were reviewed and approved by Ethics Committee of the Medical University of Lodz. The patients/participants provided their written informed consent to participate in this study.

## Author Contributions

Conceptualization, SM. Formal analysis, SM and KS. Funding acquisition, WP. Investigation, SM, KS, HJ, JM-D, AB, LG, and WP. Methodology, SM. Project administration, SM. Resources, SM, KS, JM-D, AB, LG, and WP. Writing—original draft, SM. Writing—review and editing, SM, KS, HJ, JM-D, AB, LG, and WP. All authors have read and agreed to the published version of the manuscript.

## Funding

The study was funded by the Medical University of Lodz. Study-related costs and article processing charges were defrayed from the research resources of the Department of Pneumology—account number 503/1-151-03/503-11-001-19-00.

## Conflict of Interest

SM and WP have received personal fees and travel grants from Boehringer Ingelheim and Roche. KS has received personal fees from Boehringer Ingelheim and Roche. JM-D and AB have received travel grants from Boehringer Ingelheim and Roche.

The remaining authors declare that the research was conducted in the absence of any commercial or financial relationships that could be construed as a potential conflict of interest.

## Publisher’s Note

All claims expressed in this article are solely those of the authors and do not necessarily represent those of their affiliated organizations, or those of the publisher, the editors and the reviewers. Any product that may be evaluated in this article, or claim that may be made by its manufacturer, is not guaranteed or endorsed by the publisher.
