# Supplementary material for: Longitudinal and Comparative Measures of Serum Chitotriosidase and YKL-40 in Patients With Idiopathic Pulmonary Fibrosis
Source: Front Immunol. 2022 Feb 10;13:760776. doi: 10.3389/fimmu.2022.760776 (PMC8866556; doi:10.3389/fimmu.2022.760776)
Supplement: Supplementary file 1 [file DataSheet_1.pdf]

Supplementary Table S1. Baseline correlations of serum CHIT1 and YKL-40 with clinical measures in IPF patients

	FVC (l)	FVC % pred.	$T_{L,CO}$ (mmol/min/kPa)	$T_{L,CO}$ % pred.	Age (years)	CPI score	6MWT (meters)
**CHIT1 activity [nmol/ml/h]**	r=-0.49 p=0.01	r=-0.17 p=0.42	r=-0.33 p=0.12	r=0.02 p=0.94	r=0.20 p=0.34	r=-0.15 p=0.47	r=-0.03 p=0.91
**YKL-40 [ng/ml]**	r=0.21 p=0.32	r=0.27 p=0.19	r=-0.22 p=0.29	r=-0.19 p=0.37	r=0.56 p=0.004	r=0.54 p=0.006	r=-0.35 p=0.09

Abbreviations: IPF – idiopathic pulmonary fibrosis, FVC – forced vital capacity, $T_{L,CO}$ – transfer factor of the lung for carbon monoxide, CPI – composite physiologic index, 6MWT – six-minute walk test, CHIT1 – chitotriosidase, YKL-40 – chitinase 3-like-1

Supplementary Table S2. Correlations of serum CHIT1 and YKL-40 with clinical measures in IPF patients after 6 months of antifibrotic therapy

	FVC (l)	FVC % pred.	$T_{L,CO}$ (mmol/min/kPa)	$T_{L,CO}$ % pred.	Age (years)	CPI score	6MWT (meters)
**CHIT1 activity [nmol/ml/h]**	r=-0.10 p=0.65	r=0.16 p=0.45	r=0.13 p=0.55	r=0.25 p=0.24	r=0.16 p=0.43	r=-0.08 p=0.69	r=-0.16 p=0.49
**YKL-40 [ng/ml]**	r=0.21 p=0.32	r=0.15 p=0.49	r=-0.27 p=0.19	r=-0.31 p=0.15	r=0.48 p=0.01	r=0.55 p=0.004	r=-0.45 p=0.04

Abbreviations: IPF – idiopathic pulmonary fibrosis, FVC – forced vital capacity, $T_{L,CO}$ – transfer factor of the lung for carbon monoxide, CPI – composite physiologic index, 6MWT – six-minute walk test, CHIT1 – chitotriosidase, YKL-40 –chitinase 3-like-1

Supplementary Table S3. Correlations of serum CHIT1 and YKL-40 with clinical measures in IPF patients after 12 months of antifibrotic therapy

	**FVC (l)**	**FVC % pred.**	**$T_{L,CO}$ (mmol/min/kPa)**	**$T_{L,CO}$ % pred.**	**Age (years)**	**CPI score**	**6MWT (meters)**
**CHIT1 activity [nmol/ml/h]**	r=-0.37 p=0.07	r=-0.21 p=0.31	r=-0.12 p=0.58	r=-0.11 p=0.60	r=0.14 p=0.52	r=0.01 p=0.96	r=-0.19 p=0.36
**YKL-40 [ng/ml]**	r=0.38 p=0.06	r=0.33 p=0.11	r=-0.07 p=0.74	r=-0.02 p=0.94	r=0.45 p=0.02	r=0.30 p=0.14	r=-0.22 p=0.30

Abbreviations: IPF – idiopathic pulmonary fibrosis, FVC – forced vital capacity, $T_{L,CO}$ – transfer factor of the lung for carbon monoxide, CPI – composite physiologic index, 6MWT – six-minute walk test, CHIT1 – chitotriosidase, YKL-40 –chitinase 3-like-1

Supplementary Table S4. Correlations of serum CHIT1 and YKL-40 with clinical measures in IPF patients after 18 months of antifibrotic therapy

	**FVC (l)**	**FVC % pred.**	**$T_{L,CO}$ (mmol/min/kPa)**	**$T_{L,CO}$ % pred.**	**Age (years)**	**CPI score**	**6MWT (meters)**
**CHIT1 activity [nmol/ml/h]**	r=-0.32 p=0.12	r=-0.09 p=0.68	r=-0.15 p=0.49	r=-0.004 p=0.99	r=0.10 p=0.63	r=-0.18 p=0.38	r=0.03 p=0.89
**YKL-40 [ng/ml]**	r=0.27 p=0.20	r=0.23 p=0.27	r=-0.14 p=0.51	r=-0.18 p=0.41	r=0.41 p=0.04	r=0.33 p=0.10	r=-0.32 p=0.13

Abbreviations: IPF – idiopathic pulmonary fibrosis, FVC – forced vital capacity, $T_{L,CO}$ – transfer factor of the lung for carbon monoxide, CPI – composite physiologic index, 6MWT – six-minute walk test, CHIT1 – chitotriosidase, YKL-40 –chitinase 3-like-1

Supplementary Table S5. Correlations of serum CHIT1 and YKL-40 with clinical measures in IPF patients after 24 months of antifibrotic therapy

	FVC (l)	FVC % pred.	$T_{L,CO}$ (mmol/min/kPa)	$T_{L,CO}$ % pred.	Age (years)	CPI score	6MWT (meters)
**CHIT1 activity [nmol/ml/h]**	r=-0.23 p=0.28	r=-0.19 p=0.36	r=-0.17 p=0.44	r=-0.25 p=0.25	r=-0.23 p=0.27	r=0.26 p=0.21	r=0.12 p=0.60
**YKL-40 [ng/ml]**	r=0.40 p=0.04	r=0.22 p=0.29	r=-0.06 p=0.79	r=-0.18 p=0.40	r=0.31 p=0.13	r=0.25 p=0.23	r=-0.08 p=0.72

Abbreviations: IPF – idiopathic pulmonary fibrosis, FVC – forced vital capacity, $T_{L,CO}$ – transfer factor of the lung for carbon monoxide, CPI – composite physiologic index, 6MWT – six-minute walk test, CHIT1 – chitotriosidase, YKL-40 – chitinase 3-like-1

Supplementary Table S6. Correlations of changes in serum CHIT1 activity and YKL-40 concentration levels with changes in PFTs and 6MWT over the first year of antifibrotic treatment in the stables subgroup (n=16).

	Change in FVC% pred.	Change in $T_{L,CO}$% pred.	Change in 6MWT in %
**Change in CHIT1 activity in %**	r=0.29 p=0.27	r=0.02 p=0.93	r=-0.02 p=0.95
**Change in YKL-40 concentration in %**	r=0.46 p=0.08	r=0.16 p=0.54	r=0.09 p=0.74

Abbreviations: FVC – forced vital capacity, $T_{L,CO}$ – transfer factor of the lung for carbon monoxide, 6MWT – six-minute walk test, CHIT1 – chitotriosidase, YKL-40 –chitinase 3-like-1

Supplementary Table S7. Correlations of changes in serum CHIT1 activity and YKL-40 concentration levels with changes in PFTs and 6MWT over the first year of antifibrotic treatment in the progressors subgroup (n=9).

	**Change in FVC % pred.**	**Change in $T_{L,CO}$ % pred.**	**Change in 6MWT in %**
**Change in CHIT1 activity in %**	r=-0.07<br>p=0.88	r=0.69<br>p=0.07	r=-0.17<br>p=0.70
**Change in YKL-40 concentration in %**	r=-0.13<br>p=0.74	r=0.27<br>p=0.49	r=-0.27<br>p=0.49

Abbreviations: FVC – forced vital capacity, $T_{L,CO}$ – transfer factor of the lung for carbon monoxide, 6MWT – six-minute walk test, CHIT1 – chitotriosidase, YKL-40 –chitinase 3-like-1

Supplementary Table S8. Correlations of changes in serum CHIT1 activity and YKL-40 concentration levels with changes in PFTs and 6MWT over the second year of antifibrotic treatment in the stables subgroup (n=15).

	**Change in FVC % pred.**	**Change in $T_{L,CO}$% pred.**	**Change in 6MWT in %**
**Change in CHIT1 activity in %**	r=-0.32<br>p=0.16	r=-0.22<br>p=0.36	r=0.33<br>p=0.19
**Change in YKL-40 concentration in %**	r=-0.53<br>p=0.047	r=0.08<br>p=0.80	r=-0.09<br>p=0.80

Abbreviations: FVC – forced vital capacity, $T_{L,CO}$ – transfer factor of the lung for carbon monoxide, 6MWT – six-minute walk test, CHIT1 – chitotriosidase, YKL-40 – chitinase 3-like-1

Supplementary Table S9. Correlations of changes in serum CHIT1 activity and YKL-40 concentration levels with changes in PFTs and 6MWT over the second year of antifibrotic treatment in the progressors subgroup (n=10).

	**Change in FVC % pred.**	**Change in $T_{L,CO}$ % pred.**	**Change in 6MWT in %**
**Change in CHIT1 activity in %**	r=-0.66<br>p=0.04	r=-0.45<br>p=0.23	r=0.30<br>p=0.41
**Change in YKL-40 concentration in %**	r=0.22<br>p=0.54	r=-0.37<br>p=0.34	r=-0.36<br>p=0.31

Abbreviations: FVC – forced vital capacity, $T_{L,CO}$ – transfer factor of the lung for carbon monoxide, 6MWT – six-minute walk test, CHIT1 – chitotriosidase, YKL-40 – chitinase 3-like-1

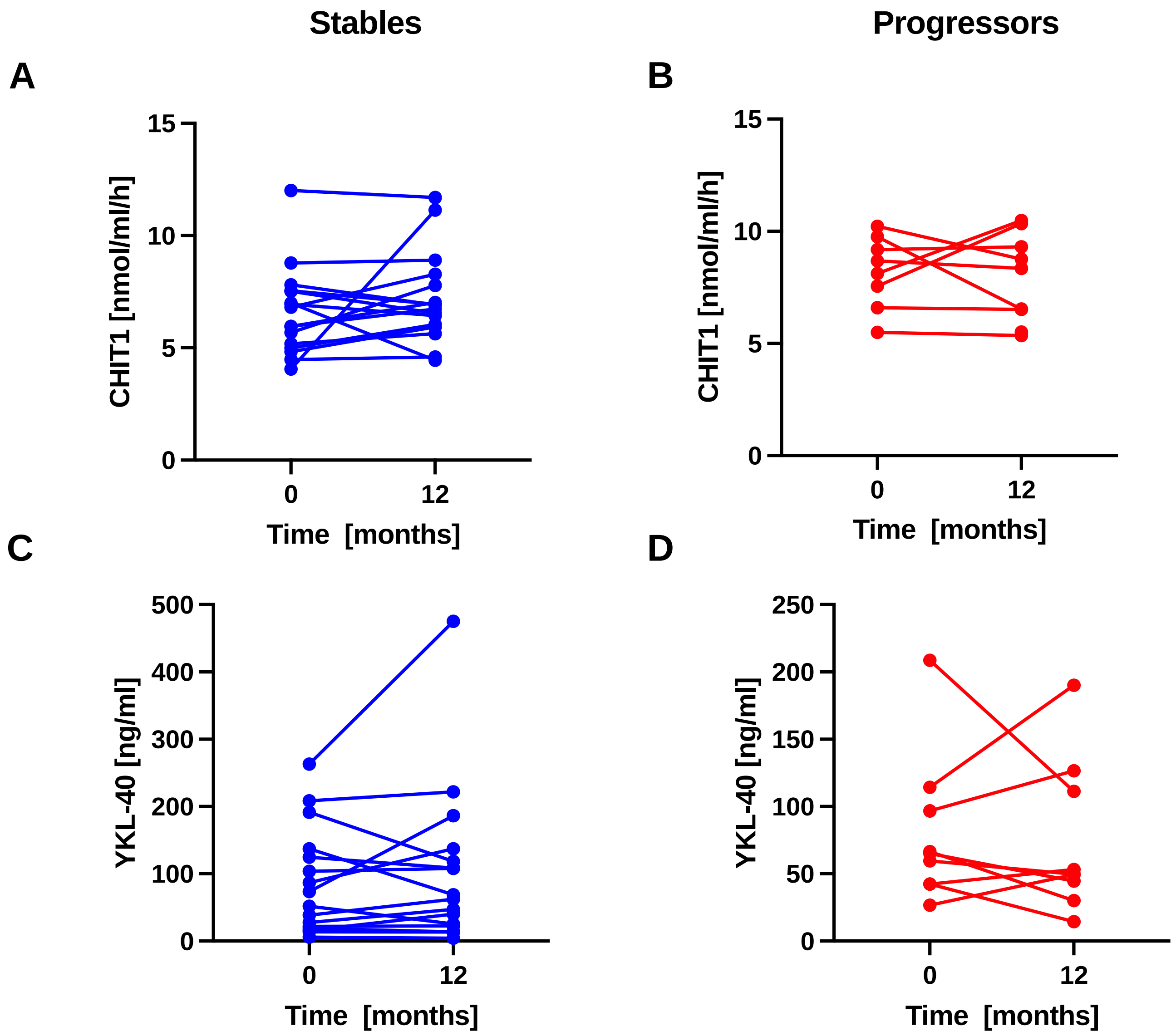

Supplementary Figure S1. Single patient dynamic changes in serum CHIT1 and YKL-40 over the first year of antifibrotic therapy in the stables (n=16) and progressors (n=9) subgroups of patients with IPF.

Notes: Panels showing dynamic changes of: (**A**) serum CHIT1 activity levels in the stables subgroup, (**B**) serum CHIT1 activity levels in the progressors subgroup, **(C)** serum YKL-40 concentration levels in the stables subgroup, **(D)** serum YKL-40 concentration levels in the progressors subgroup,

Abbreviations: CHIT1 – chitotriosidase, YKL-40 – chitinase 3-like-1

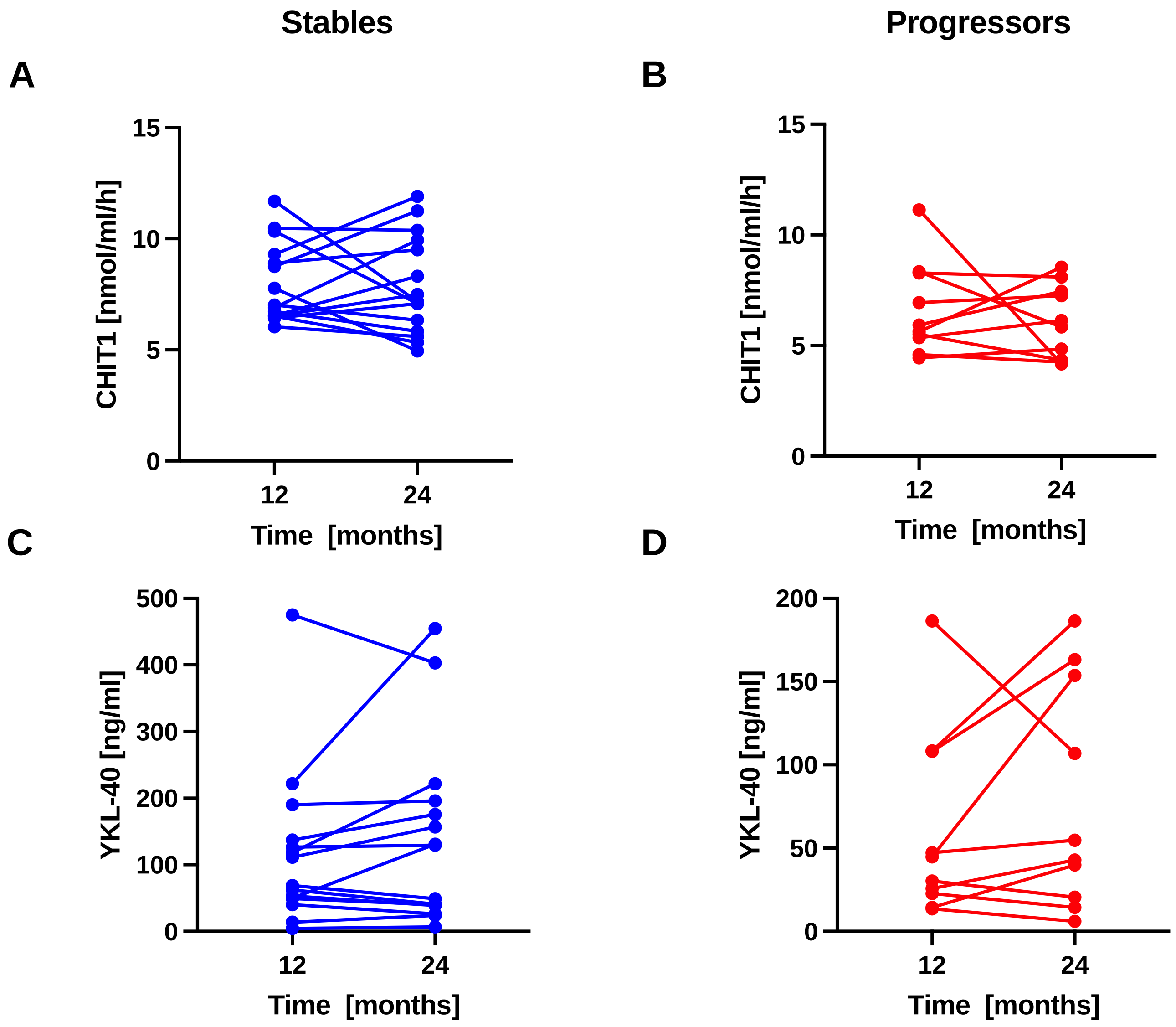

Supplementary Figure S2. Single patient dynamic changes in serum CHIT1 and YKL-40 over the second year of antifibrotic therapy in the stables (n=15) and progressors (n=10) subgroups of patients with IPF.

Notes: Panels showing dynamic changes of: (**A**) serum CHIT1 activity levels in the stables subgroup, (**B**) serum CHIT1 activity levels in the progressors subgroup, **(C)** serum YKL-40 concentration levels in the stables subgroup, **(D)** serum YKL-40 concentration levels in the progressors subgroup,

Abbreviations: CHIT1 – chitotriosidase, YKL-40 –chitinase 3-like-1
